# *Yersinia pestis* insecticidal-like toxin complex (Tc) family proteins: characterization of expression, subcellular localization, and potential role in infection of the flea vector

**DOI:** 10.1186/1471-2180-12-296

**Published:** 2012-12-18

**Authors:** Justin L Spinner, Clayton O Jarrett, Doris L LaRock, Samuel I Miller, Carleen M Collins, B Joseph Hinnebusch

**Affiliations:** 1 Laboratory of Zoonotic Pathogens, Rocky Mountain Laboratories, NIAID, NIH, Hamilton, MT, 59840, USA; 2 Department of Microbiology, University of Washington, Seattle, WA, 98195, USA; 3 Department of Genome Sciences, Immunology, and Medicine, University of Washington, Seattle, WA, 98195, USA

**Keywords:** *Yersinia pestis*, Toxin complex proteins, YitA, YipA, YitR, *Xenopsylla cheopis*

## Abstract

**Background:**

Toxin complex (Tc) family proteins were first identified as insecticidal toxins in *Photorhabdus luminescens* and have since been found in a wide range of bacteria. The genome of *Yersinia pestis*, the causative agent of bubonic plague, contains a locus that encodes the Tc protein homologues YitA, YitB, YitC, and YipA and YipB. Previous microarray data indicate that the Tc genes are highly upregulated by *Y. pestis* while in the flea vector; however, their role in the infection of fleas and pathogenesis in the mammalian host is unclear.

**Results:**

We show that the Tc proteins YitA and YipA are highly produced by *Y. pestis* while in the flea but not during growth in brain heart infusion (BHI) broth at the same temperature. Over-production of the LysR-type regulator YitR from an exogenous plasmid increased YitA and YipA synthesis in broth culture. The increase in production of YitA and YipA correlated with the *yitR* copy number and was temperature-dependent. Although highly synthesized in fleas, deletion of the Tc proteins did not alter survival of *Y. pestis* in the flea or prevent blockage of the proventriculus. Furthermore, YipA was found to undergo post-translational processing and YipA and YitA are localized to the outer membrane of *Y. pestis*. YitA was also detected by immunofluorescence microscopy on the surface of *Y. pestis.* Both YitA and YipA are produced maximally at low temperature but persist for several hours after transfer to 37°C.

**Conclusions:**

*Y. pestis* Tc proteins are highly expressed in the flea but are not essential for *Y. pestis* to stably infect or produce a transmissible infection in the flea. However, YitA and YipA localize to the outer membrane and YitA is exposed on the surface, indicating that at least YitA is present on the surface when *Y. pestis* is transmitted into the mammalian host from the flea.

## Background

*Yersinia pestis*, the causative agent of bubonic plague, is maintained in nature by flea-rodent enzootic cycles and incidentally transmitted to humans through the bite of an infected flea. Like *Y. pestis*, the closely related *Yersinia pseudotuberculosis* and the more distantly related *Yersinia enterocolitica* harbor a virulence plasmid that encodes a type III secretion system (T3SS) and effector proteins (Yops). However, *Y. pseudotuberculosis* and *Y. enterocolitica* are not transmitted by fleas and cause enteric disease in humans [1-3]. Several *Y. pestis* genes have been found to be required to infect and be transmitted by fleas. These include the murine toxin gene (*ymt)*, the hemin storage (*hmsHFRS*) genes, the diguanylate cyclases encoded by *y3730* and *hmsT*, and *gmhA*. The *y3730, hms,* and *gmhA* genes are needed for bis-(3^′^-5^′^)-cyclic dimeric GMP (c-di-GMP) metabolism, formation of an extracellular polysaccharide and a lipopolysaccharide core modification, respectively, that are necessary for biofilm formation and blockage of the flea proventriculus [4-7]. The murine toxin (*ymt*) gene, which encodes a phospholipase D, is required for survival of *Y. pestis* within the flea midgut [8]. However, additional genes may also be important for survival and replication of *Y. pestis* within the flea or play a role in transmission to and survival within the mammalian host.

Recent microarray data indicate that a number of genes are differentially regulated by *Y. pestis* during infection of the flea compared to *in vitro* culture at the same temperature [9]. Among these were a group of upregulated genes that share homology with insect toxin genes of the Toxin complex (Tc) family. First identified in *Photorhabdus luminescens*, which maintains a symbiotic relationship with entomopathogenic nematodes of the family Heterorhabditidae [10,11], Tc protein homologues are also found in a number of other bacteria including *Y. enterocolitica* and *Y. pseudotuberculosis*[12]. In *P*. *luminescens*, Tc genes are found at four loci which have a high degree of similarity and can be grouped into three basic genetic elements (*tcdA*/*tcaAB*/*tccAB* [type A], *tcdB*/*tcaC* [type B], and *tccC* [type C]) [11]. The *P. luminescens* toxins are upregulated in the insect host [13], interact with each other to form large active toxin complexes and are highly insecticidal [14,15]. Furthermore, they have been shown to disrupt the actin cytoskeleton of NIH 3T3 Swiss mouse fibroblast cells [15,16]. More recently, *P. luminescens* toxin complexes were found to ADP-ribosylate actin and Rho GTPases, respectively, which caused actin polymerization and clustering in human HeLa cells and resulted in altered phagocytosis by *Galleria mellonella* hemocytes [17].

**Figure 1:**
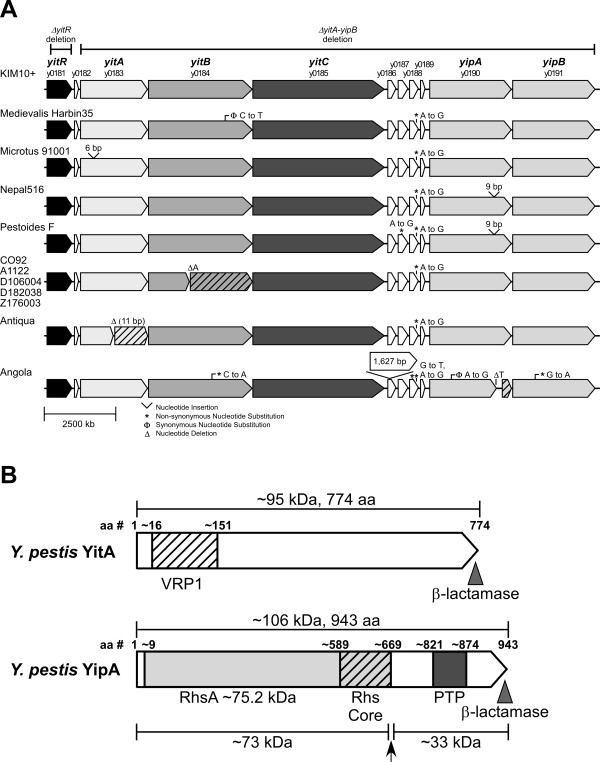
**A) The Tc protein locus of *Y. pestis *contains the *yitABC *and *yipAB *insecticidal-like protein genes and the upstream regulator *yitR*.** Alignment of the Tc locus for all sequenced *Y. pestis* strains is shown with differences from KIM10+ indicated. The deletions in the *Y. pestis* KIM6+Δ*yitR* and Δ*yitA-yipB* mutant strains used in this study are indicated. **B**) Domain structure of YitA and YipA. Hatch marks represent the region of YitA with similarity to the *Salmonella* virulence plasmid A (VRP1) protein family. The light gray area designates the region of YipA similar to the Rhs protein family. Light gray shaded hatch marks indicate the RHS repeat-associated core domain. Dark gray represents the region sharing homology to the protein tyrosine phosphatase (PTP) protein family and the PTP catalytic domain. The arrow indicates the inferred location of post-translational processing of YipA. The translational fusion junction of the full-length YitA and YipA with the mature β-lactamase is designated by shaded triangles.

Tc protein homologues are found in all sequenced *Y. pestis* strains available to date (Figure 1A). *Y. pestis* Tc proteins are termed YitA (TcaA-like), YitB (TcaB-like), YitC (TcaC-like), and YipA and YipB (TccC- like) and are found within a single locus in the chromosome (Figure 1A) [18]. Although their sequences are highly conserved, *Y. pestis* strains CO92, A1122, D106004, D182038, and Z176003 have an apparent frameshift mutation in *yitB* (missing a single adenosine [A] from a string of seven A’s), and strain Antiqua has an eleven nucleotide deletion resulting in a frameshift mutation in *yitA*. Additionally, *Y. pestis* Angola has a frameshift mutation in the C-terminus of *yipA* (Figure 1A). Previous studies have indicated that the Tc genes undergo thermoregulation, with greater expression after growth at 21°C or 26°C than 37°C [18-20]. Reverse transcription polymerase chain reaction data indicate that *yitA*, -*B, -C* genes form an operon and *yipA*, -*B* genes are on a different transcriptional unit [18]. Deletion of the upstream LysR-like regulator (*yitR*) decreased the production of Tc proteins [18], indicating that YitR, which is also upregulated following growth of *Y. pestis* in the flea [9], is a positive regulator of expression. Similarly to *P*. *luminescens*, *Y. pestis* Tc proteins form a large multicomponent protein complex that contains all 5 Tc proteins [18]. Complex formation requires YitA and YitB, and YitC is necessary for association of YipA and YipB with the complex [18].

Although there is no defined biological role for the *Yersinia* Tc proteins, functional studies indicate that they are important in the interaction with insect cells or specific mammalian host cells. *Y. pestis* Tc proteins are not toxic to *M. sexta*[16], whereas *Y. pseudotuberculosis* and *Y. enterocolitica* (biotype 2–5, including strain W22703) Tc proteins are toxic, although they are much less potent than *P. luminescens* toxins [12,21,22]. Whereas *P*. *luminescens* toxins are also toxic to *Xenopsylla cheopis* rat fleas, *Y. pestis* and *Y. pseudotuberculosis* Tc proteins are not [2]. Additionally, *Y. pseudotuberculosis* and *Y. pestis* Tc proteins are not active against *Spodoptera frugiperda* (Sf9) insect cells [16]. However, unlike *Y. pseudotuberculosis*, *Y. pestis* Tc proteins are active against NIH 3T3 mouse fibroblast cells but not Caco-2 human intestinal epithelial cells [16], indicating specificity for certain host environments. There is evidence for T3SS-dependent translocation of *Y. pestis* Tc proteins into host cells [18] and Tc genes (*yitA, -B, -C*) are upregulated within J774A.1 macrophages [23]. Furthermore, repression of Tc gene production (−Δ*yitR*) increased phagocytosis by macrophages of *Y. pestis* isolated from fleas [9]. However, actual levels of the *Y. pestis* Tc proteins in the flea or during growth in liquid culture, or a potential role in survival within or transmission from the flea have not yet been determined.

In this study, we show that the Tc proteins YitA and YipA are highly produced by *Y. pestis* in the flea but not during growth in culture at the same temperature (22°C) and that over-production of YitR increases YitA and YipA synthesis *in vitro*. YitA and YipA production was greatest during growth at lower temperatures (less than 22°C) and minimally produced at 37°C, although the proteins persisted for more than 9 hours after a transition from 22°C to 37°C. YipA appears to be processed near the C-terminus between the RhsA and PTP domains. Furthermore, YitA and YipA are localized to the outer membrane, and YitA is surface-exposed. We also show that the *Y. pestis* Tc proteins do not play a detectable role in *X. cheopis* infection or the ability to produce a transmissible infection.

## Results

### YitA and YipA are synthesized in the flea but not *in vitro* unless the YitR regulator is over-produced

**Figure 2:**
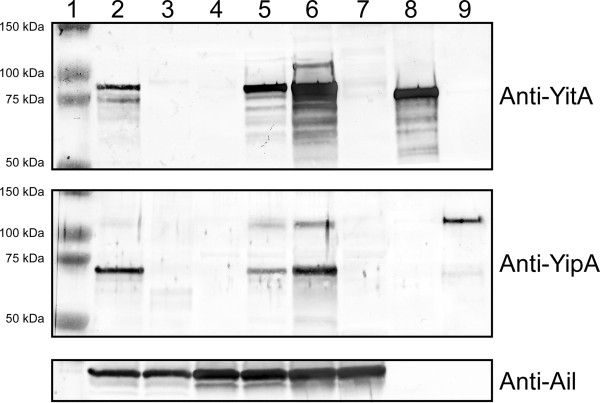
**YitA and YipA are only detectable in *Y. pestis *isolated from fleas but over-production of YitR increases their synthesis *in vitro*.** Lane 1, molecular weight ladder. Lane 2, *Y. pestis* KIM6+ isolated from infected fleas. Lane 3, KIM6+Δ*yitA-yipB* isolated from infected fleas. Lane 4, KIM6+ grown at 22°C in BHI. Lane 5, KIM6+ (pWKS130::*yitR*) grown at 22°C in BHI. Lane 6, KIM6+ (pCR-XL-TOPO::*yitR*) grown at 22°C in BHI. Lane 7, KIM6+Δ*yitA-yipB* (pCR-XL-TOPO::*yitR*) grown at 22°C in BHI. Lanes 8–9, recombinant YitA and YipA purified from *E. coli*. Panels show Western blots probed with anti-YitA, anti-YipA, or anti-Ail (sample loading control) antiserum.

A diagram of the *Y. pestis* Tc locus is shown in Figure 1a. *X. cheopis* fleas were infected with KIM6+ or KIM6+Δ*yitA-yipB* (Figure 1A) to compare YitA and YipA (Figure 1B) protein levels following growth in the flea to growth in BHI culture. YitA and YipA were both highly produced by *Y. pestis* in the flea (Figure 2, lane 2) compared to stationary phase BHI cultures (Figure 2, lane 4) incubated at 22°C, the same temperature at which the fleas were maintained. YitA was detected as a prominent band around 95 kDa, which corresponded to the expected size based on the YitA amino acid sequence. YipA was detected as two major bands. The smaller band at ~73 kDa was the most prominent. The larger band at ~106 kDa corresponds to the full length YipA predicted by its amino acid sequence and with recombinant YipA synthesized in and purified from *E. coli* (Figure 2, lane 9).

To determine if over-production of YitR would result in increased levels of YitA and YipA proteins during growth *in vitro*, the regulator *yitR* was cloned with its native promoter into the low-copy plasmid pWKS130 and the high-copy plasmid pCR-XL-TOPO. *Y. pestis* KIM6+ carrying pWKS130::*yitR* (Figure 2, lane 5) or pCR-XL-TOPO::*yitR* (Figure 2, lane 6) had increased levels of YitA and YipA proteins following growth in BHI at 22°C compared to wild-type KIM6+ (Figure 2, lane 4) and KIM6+Δ*yitA-yipB* (pCR-XL-TOPO::*yitR*) (Figure 2, lane 7). YitA and YipA protein increased with an increase in *yitR* copy number (Figure 2, lanes 5–6). The sizes of the YitA and YipB protein produced by all the strains under environmental conditions were similar (Figure 2, lanes 2, 5, 6). No detectable YitA or YipA protein was produced by the KIM6+Δ*yitR* deletion mutant (data not shown).

### *In vitro* production of YitA and YipA by *Y. pestis* is dependent on growth temperature but not on culture medium

**Figure 3:**
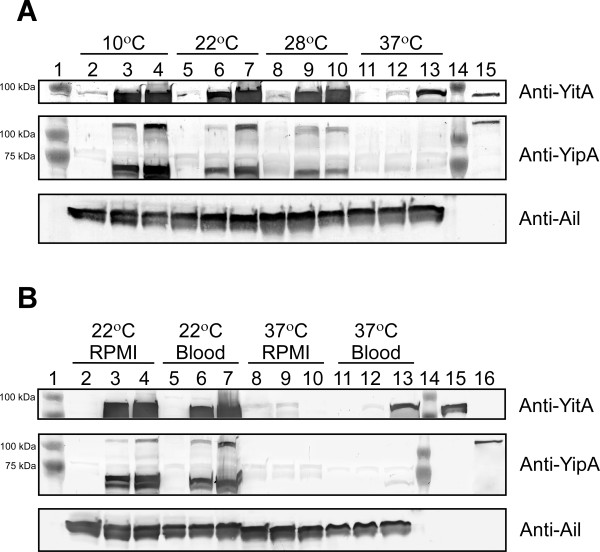
**Maximal synthesis of YitA and YipA during growth at low temperatures. A**) KIM6+ (lanes 2, 5, 8, and 11), KIM6+ (pWKS130::*yitR*) (lanes 3, 6, 9, and 12) and KIM6+ (pCR-XL-TOPO::*yitR*) (lanes 4, 7, 10, and 13) grown overnight at 10°C, 22°C, 28°C or 37°C in BHI broth. YitA and YipA purified from *E. coli* (lane 15). **B**) KIM6+ (lanes 2, 5, 8, and 11), KIM6+ (pWKS130::*yitR*) (lanes 3, 6, 9, and 12) and KIM6+ (pCR-XL-TOPO::*yitR*) (lanes 4, 7, 10, and 13) grown overnight at 22°C or 37°C in either RPMI 1640 (RPMI) or whole sheep blood (Blood). YitA and YipA purified from *E. coli* (lanes 15 and 16). Panels show Western blots probed with anti-YitA, anti-YipA, or anti-Ail (sample loading control) antiserum.

*Y. pestis* KIM6+, KIM6+ (pWKS130::*yitR*), and KIM6+ (pCR-XL-TOPO::*yitR*) were grown in BHI at 10°C, 22°C, 28°C, or 37°C overnight to determine YitA and YipA synthesis at different growth temperatures. YitA production in parental KIM6+ was detected after growth at 10°C (Figure 3A, lane 2). Full-size YipA was not detected in KIM6+ at any temperature (Figure 3A, lanes 2, 5, 8, and 11). When plasmid pWKS130::*yitR* was present, YitA was seen at all temperatures, with the maximum level at 10°C; the level decreased when the growth temperature was 37°C (Figure 3A, lanes 3, 6, 9, and 12). When plasmid pWKS130::*yitR* was present, YipA production was also greatest after growth at 10°C (Figure 3A, lane 3) and decreased when the growth temperature was 37°C (Figure 3A, lanes 6, 9, and 12); however, very little was seen at 37°C and the larger molecular weight band was no longer present (Figure 3A, lane 12). *Y. pestis* KIM6+ with the high-copy number pCR-XL-TOPO::*yitR* had the greatest production of YitA and YipA, which also decreased when the growth temperature was 37°C (Figure 3A, lanes 4, 7, 10, and 13). For each of the strains tested, levels of YitA and YipA were comparable after growth at 22°C or 28°C (Figure 3A, lanes 5, 6, 7, 8, 9 and 10).

YitA and YipA production following growth in both blood and RPMI 1640 was equivalent to production following growth in BHI. YitA and YipA were produced to the greatest extent after growth at 22°C in RPMI 1640 and blood (Figure 3B, lanes 2–7) and levels dramatically decreased following growth at 37°C (Figure 3B, lanes 8–12). As with growth in BHI, *Y. pestis* with the high-copy pCR-XL-TOPO::*yitR* had the greatest level of production (Figure 3B, lanes 4, 7, 10, and 13) compared to KIM6+ with the low-copy YitR plasmid and wild-type KIM6+.

### YitA and YipA persist for several hours following a growth temperature shift to 37°C

**Figure 4:**
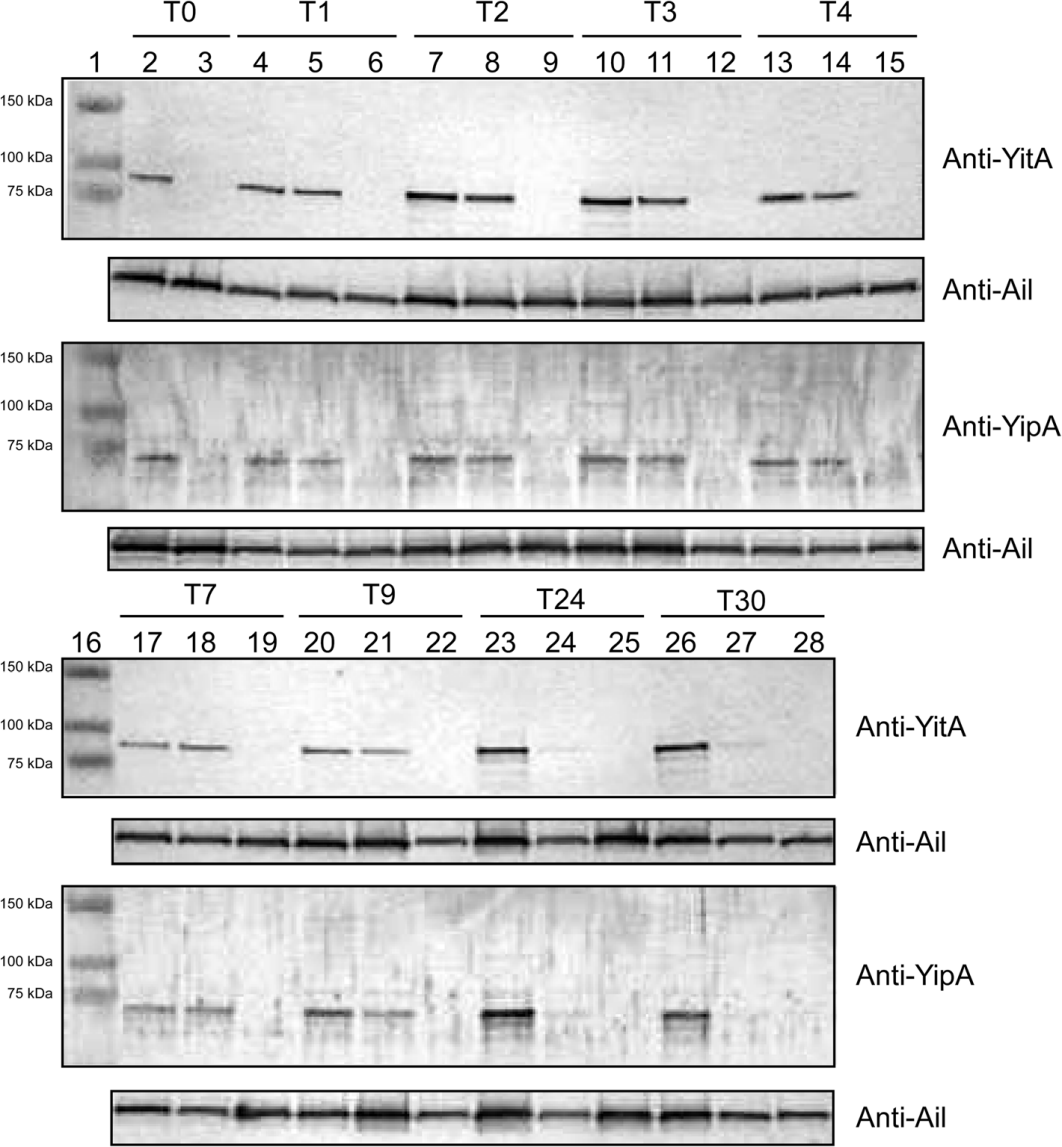
**YitA and YipA proteins persist in *Y. pestis *for at least 9 hours after transfer to 37°C.***Y. pestis* KIM6+ (pCR-XL-TOPO::*yitR*) (lanes 5, 8, 11, 14, 18, 21, 24, and 27) and KIM6+Δ*yitA-yipB* (pCR-XL-TOPO::*yitR*) (lanes 3, 6, 9, 12, 15, 19, 22, 25, and 28) were grown overnight at 22°C and subsequently transferred to 37°C for the indicated amount of time prior to sample collection. A matched KIM6+ (pCR-XL-TOPO::*yitR*) was maintained at 22°C as a positive reference control (lanes 2, 4, 7, 10, 13, 17, 20, 23, and 26). T0 = initial time point, T(x) = x hours at 37°C. Panels show Western blots probed with anti-YitA, anti-YipA, or anti-Ail (sample loading control) antiserum.

YitA and YipA levels over time following a temperature shift from 22°C to 37°C was determined by Western blot analysis. YitA and YipA synthesized by KIM6+ (pCR-XL-TOPO::*yitR*) following growth in BHI at 22°C were still present 7 hours after an upshift to 37°C (Figure 4, lanes 5, 8, 11, 14, and 18). After 9 hours, a slight reduction in YitA and YipA protein was seen in the 37°C culture compared to the matched culture maintained at 22°C (Figure 4, lanes 21 and 20, respectively). After 24 hours, there was a significant decrease in detectable YitA and YipA (Figure 4, lane 24) in the 37°C culture. After 30 hours at 37°C, only a small quantity of YitA remained and no detectable YipA (Figure 4, lane 27).

### Evidence for post-translational processing of YipA

**Figure 5:**
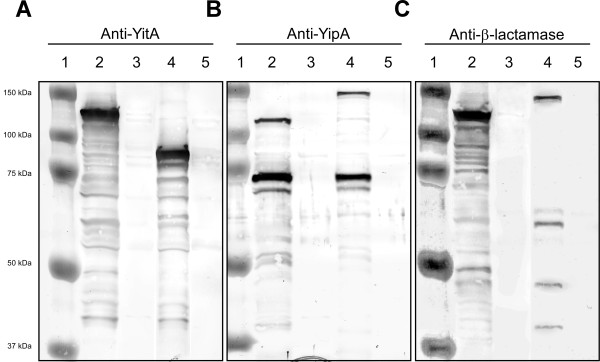
**Characterization of post-translational processing of YipA.** KIM6+ (pCR-XL-TOPO::*yitR*) with the C-terminus of YitA (Lane 2) or YipA (Lane 4) tagged with mature β-lactamase were grown overnight at 22°C in BHI broth. KIM6+ YitA-β-lactamase (Lane 3) or YipA-β-lactamase (Lane 5) not over-producing YitR grown overnight at 22°C in BHI broth are provided as negative controls. Panels show Western blots probed with **A**) anti-YitA, **B**) anti-YipA, or **C**) anti- β-lactamase antiserum

Two forms of YipA were typically detected by Western blot: the predicted full-length protein at ~106 kDa and a smaller protein of ~73 kDa, with the smaller form often predominating (Figures 2, 3, 4). To determine which of the bands detected using anti-YipA serum correspond to the N-terminal region and the C-terminal region of YipA, *Y. pestis* strains containing translational fusions of mature β-lactamase (~28.9 kDa) to the C-terminus of YitA or YipA were constructed (Figure 1B). After overnight growth at 22°C, *Y. pestis* YitA-β-lactamase and YipA-β-lactamase with or without plasmid pCR-XL-TOPO::*yitR* were assayed by Western blot. YitA-β-lactamase was detected by anti-YitA serum as a single band at ~123 kDa (due to the addition of the mature β-lactamase) with a light smear of smaller bands (Figure 5A, lane 2), whereas wild-type YitA was detected around 95 kDa (Figure 5A, lane 4). Anti-β-lactamase antibody also detected full length YitA-β-lactamase at ~123 kDa as a prominent band and a smear of several smaller bands (Figure 5C, lane 2).

Anti-YipA serum detected YipA-β-lactamase as two prominent bands. The YipA-β-lactamase lower band at ~73 kDa (Figure 5B, lane 4) was the same size as the lower band seen with wild-type YipA (Figure 5B, lane 2). The upper band of YipA-β-lactamase was detected at ~135 kDa (Figure 5B, lane 4), whereas the upper band of wild-type YipA was detected at ~106 kDa (Figure 5B, lane 2). Anti-β-lactamase antibody detected the upper ~135 kDa band corresponding to full-length YipA-β-lactamase (Figure 5C, lane 4). However, the lower ~73 kDa band was not detected by anti-β-lactamase antibody (Figure 5C, lane 4); although a distinct band at ~62 kDa was detected by anti-β-lactamase antibody (Figure 5C, lane 4). This indicates that the YipA molecular weight band detected by anti-YipA at ~73 kDa (Figure 5B, lane 4) represents the N-terminus of YipA, whereas the smaller molecular weight band detected by anti-β-lactamase antibody (~62 kDa) represents the C-terminal region of YipA fused to β-lactamase (Figure 5C, lane 4).

### YitA and YipA are localized in the outer membrane of *Y. pestis*

**Figure 6:**
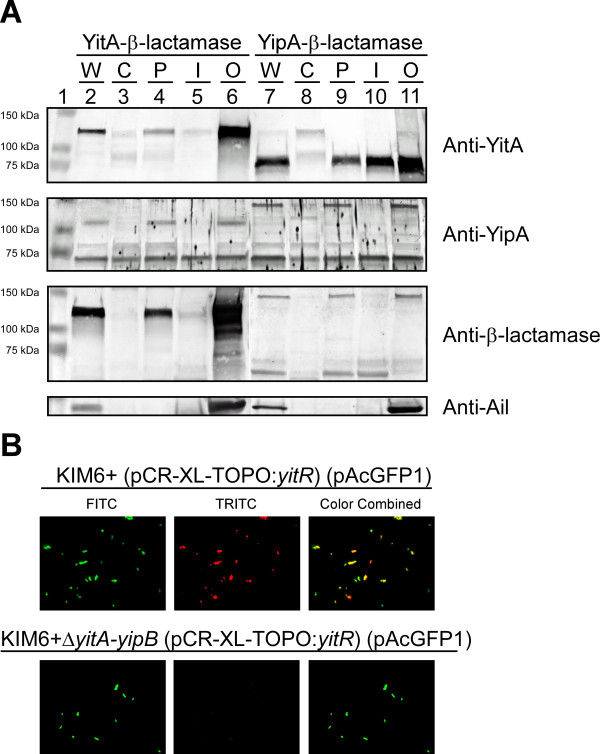
**YitA and YipA are localized to the outer membrane fraction of *Y. pestis *and YitA is detectable on the surface of the bacteria.A**) *Y. pestis* KIM6+ (pCR-XL-TOPO::*yitR*) YitA-β-lactamase (Lanes 2–6) or YipA-β-lactamase (Lanes 7–11) grown overnight at 22°C in BHI were lysed and separated into cytoplasmic (C), periplasmic (P), cytosolic inner membrane (I), and outer membrane (O) fractions and analyzed by Western blot. Whole cell lysates (W) are provided as a control for both strains. Panels show Western blots probed with antisera to YitA, YipA, and β-lactamase, or Ail (a known *Y. pestis* outer membrane protein). **B**) Evidence of surface exposed YitA on *Y. pestis*. The top panel includes images of *Y. pestis* KIM6+ (pCR-XL-TOPO::*yitR*) (pAcGFP1, fluoresces green) grown overnight at 22°C in BHI. YitA was detected by incubating fixed bacteria with anti-YitA serum and staining with Alexa Fluor 568 goat anti-rabbit IgG (fluoresces red). Fluorescence was imaged under green (FITC) and red (TRITC) filters, artificially colored, and merged. Images of KIM6+Δ*yitA-yipB* (pCR-XL-TOPO::*yitR*) (pAcGFP1) prepared and imaged by the same method are shown in the bottom row as a background control

To determine where YitA and YipA are localized within *Y. pestis*, cytoplasmic, periplasmic, inner membrane and outer membrane fractions were collected from KIM6+ YitA-β-lactamase (pCR-XL-TOPO::*yitR*) and KIM6+ YipA-β-lactamase (pCR-XL-TOPO::*yitR*) grown in BHI overnight at 22°C. YitA-β-lactamase was detected by anti-YitA (Figure 6a, top panel) and anti-β-lactamase (Figure 6A, bottom panel) antibodies predominately in the outer membrane fraction (Figure 6A, lane 6) and to a lesser extent in the periplasm (Figure 6A, lane 4). Wild-type YitA was detected in the cytoplasmic, periplasmic, inner membrane and outer membrane fractions of KIM6+ YipA-β-lactamase (Figure 6A, lanes 8–11).

Full length YipA-β-lactamase was detected by anti-YipA (Figure 6A, middle panel) and anti-β-lactamase antibodies predominately in the periplasm and outer membrane fractions (Figure 6A, lanes 9 and 11) whereas the smaller (~73 kDa) YipA band was only detected by anti-YipA serum and was present in all of the fractions at approximately the same concentration (Figure 6A, lanes 8–11). Similarly, full-length wild-type YipA was detected by anti-YipA serum primarily in the periplasm and outer membrane fractions (Figure 6A, lanes 4 and 6), with the smaller (~73 kDa) band present in all the fractions of KIM6+ YitA-β-lactamase (Figure 6A, lanes 3–6). Interestingly, the smaller (~62 kDa) YipA β-lactamase band detected by anti-β-lactamase antibodies was predominately in the periplasm and inner membrane fractions (Figure 6A, lanes 9 and 10) and only minimally present in the cytoplasm and outer membrane fractions of KIM6+ YipA-β-lactamase (Figure 6A, lanes 8 and 11). Ail, a known outer membrane protein, was used as a loading and fractionation validation control and, as expected, was detected predominately in the outer membrane fractions of both bacterial strains. Thus, although YitA and YipA were detected in all of the fractions, the full length proteins are predominately localized within the periplasm and the outer membrane fractions. Conversely, the N-terminus of processed YipA (~73 kDa) appears equally in all fractions and some quantity of the C-terminal region of YipA-β-lactamase (~62 kDa) may be retained within the inner membrane fraction.

Immunofluorescence microscopy detected YitA on the surface of paraformaldehyde fixed KIM6+ (pCR-XL-TOPO::*yitR*) (pAcGFP1) (Figure 6B, top row) but not on the surface of KIM6+Δ*yitA-yipB* (pCR-XL-TOPO::*yitR*) (pAcGFP1) (Figure 6B, bottom row). YipA could not be detected above background levels on the surface of KIM6+ (pCR-XL-TOPO::*yitR*) (pAcGFP1) using anti-YipA serum (data not shown).

### Evaluation of the role of Tc proteins during *Y. pestis* flea infection

To determine if the *Y. pestis* Tc proteins are important for survival within the flea or are required to produce a transmissible infection, we infected *X. cheopis* fleas with KIM6+ or KIM6+Δ*yitA-yipB.* In different experiments, fleas were fed on blood containing a low infectious dose (~1 x 10^7^ CFU) or a high infectious dose (~1 x 10^8^ CFU) of KIM6+ or KIM6+Δ*yitA-yipB* per mL and were maintained for 4 weeks. As expected, infection rates and the incidence of proventricular blockage increased with the number of bacteria in the infectious blood meal, but there were no differences in these rates between fleas infected with KIM6+ or with KIM6+Δ*yitA-yipB* (Table 1). The average bacterial load per infected flea was also similar for the two strains. Thus, although highly produced in the flea gut, the *Y. pestis* Tc proteins do not appear to be necessary to establish a normal transmissible infection in the insect vector.

**Table 1:** **Flea infection results with KIM6+ and KIM6+Δ*yitA-yipB***

**Strain**	**CFU/mL in blood meal**	**CFU/infected flea**^***a***^			**% Fleas infected**^***b***^			**% Fleas blocked**^***c***^
		**Day 0**	**Day 7**	**Day 28**	**Day 0**	**Day 7**	**Day 28**	
KIM6+	1.04e7	3.91e3 ± 6.45e2	1.84e5 ± 3.51e4	3.79e5 ± 4.82e4	100.0	85.0	85.0	29.0
KIM6+Δ*yitA-yipB*	1.75e7	5.95e3 ± 1.03e3	2.61e5 ± 6.40e4	4.24e5 ± 6.86e4	100.0	75.0	80.0	33.0
KIM6+	5.20e7	1.66e4 ± 2.00e3	6.16e5 ± 1.21e5	4.99e5 ± 1.00e5	100.0	95.0	80.0	49.0
KIM6+Δ*yitA-yipB*	1.55e8	4.16e4 ± 3.82e3	5.30e5 ± 1.12e5	4.75e5 ± 1.13e5	100.0	80.0	75.0	51.0

*a* Mean ± standard error of CFU counts from 20 individual female fleas collected on the indicated day after infection.

*b* Percentage of 20 female fleas collected on the indicated day after infection from which *Y. pestis* CFU were recovered.

*c* Percentage of fleas that became blocked during the 28 days after infection.

## Discussion

In this study, we show that YitA and YipA proteins are highly produced by *Y. pestis* isolated from the flea vector *X. cheopis* but not by *Y. pestis* grown *in vitro* unless the positive regulator *yitR* is over-expressed (Figure 2). This is consistent with microarray data showing a 6–50 fold increase in Tc gene expression in the flea, compared to *Y. pestis* grown in culture at the same temperature [2,9]. Previous data showed that deletion of *yitR* reduced Tc protein synthesis [18]. Additionally, expression of *yitR* is also upregulated in the flea [9]. Thus, we added *yitR* to *Y. pestis* on a low-copy and a high-copy plasmid, and found that the greatest levels of YitA and YipA were seen when *yitR* was present on the high-copy number plasmid (Figure 2). Furthermore, consistent with previous quantitative real-time polymerase chain reaction results [9], we found that deletion of *yitR* dramatically reduced YitA and YipA levels after growth in the flea (data not shown). This validates the premise that YitR acts as a positive regulator of *yitA* and *yipA* expression *in vivo*. Since YitA and YipA were not detected in culture-grown *Y. pestis* KIM6+ and collection of sufficient bacteria from fleas for multiple experiments is not feasible, the use of YitR over-producing strains were used judiciously to further study YitA and YipA.

*Y. pseudotuberculosis* Tc proteins were preferentially produced after growth at 28–37°C but not at 15°C [16]. *Y. pestis* Tc proteins have also been shown to be produced after growth at 30°C [18]. However, microarray data indicate that *Y. pestis* Tc *yit* genes are preferentially transcribed at 21°C or 26°C and down-regulated (3-fold for *yitA* and 4.2-fold for *yitR*) after growth at 37°C [19,20]. This thermoregulation is also seen with *Y. enterocolitica* W22703 Tc genes, which show a preference for low-temperature expression and have markedly down-regulated expression at 37°C [22]. Correspondingly, our data show that YitA and YipA are both thermoregulated in KIM6+, with the greatest production during growth at lower temperatures and only minimal production during growth at 37°C (Figure 3A). Furthermore, YitA and YipA underwent similar thermoregulation after growth in both RPMI 1640 and blood (Figure 3B.). Thus, YitA and YipA would not be expected to play a role in *Y. pestis* pathogenesis late in the course of mammalian infection. This is supported by gene expression data from *Y. pestis* isolated from rat bubos that show no detectable expression of *yitR*, and ~2-25 fold less expression of y*itA, B, C* and *yipB* than *Y. pestis* isolated from fleas [9,20,24]. However, *yitA,-B,-C* were all found to be upregulated 1.3- to 7.6-fold by *Y. pestis* within J774A.1 macrophage-like cells compared to bacteria grown in cell culture medium under the same conditions [23], indicating that the optimum environment for Tc protein production at 37°C may be within host phagocytes.

Western blot analysis of YitA and YipA proteins from *Y. pestis* reveals potential processing of YipA (Figure 2 and 3). YipA was consistently detected by anti-YipA serum as two distinct protein bands of ~106 kDa and ~73 kDa (Figure 2). From the amino acid sequence, YipA is predicted to be ~106 kDa. Thus, YipA may be present as a full-length protein and a processed variant. We show that an anti-β-lactamase antibody only detected the ~135-kDa full-length YipA-β-lactamase protein but not the lower weight band expected at ~102 kDa (73 kDa + 29 kDa) (Figure 5). This indicates that the 73-kDa band detected with anti-YipA serum is the N-terminus of the processed YipA. In support of this, the anti-β-lactamase antibody also detected a prominent smaller band which migrated a little over half the distance between 50 and 75 kDa at ~62 kDa. This band would correspond with the cleaved C-terminus of YipA (~33 kDa) bound to β-lactamase (29 kDa). Although both YipA bands were consistently seen in repeat experiments, there were smaller variable bands and smearing often seen using anti-YipA antibody and anti-β-lactamase antibodies. This suggests that the processed YipA is not stable and may undergo degradation under our assay conditions. The processed state of these proteins under natural conditions is difficult to explore due to limitations in the collection of bacteria from fleas. Nonetheless, the N and C-terminal regions of YitA and YipA contain predicted domains (Figure 1B). The N-terminus of YitA contains a domain that shares similarity with the *Salmonella* virulence plasmid A (VRP1) protein family. The YipA amino acid sequence indicates two conserved domains, including an N-terminus that shares similarity with the Rhs protein family reported in cell envelope biogenesis and outer membrane proteins. The YipA RhsA domain is predicted to be approximately 75.4 kDa, which corresponds to the N-terminal band of YipA at ~73 kDa. In addition, the YipA C-terminus contains a single predicted protein tyrosine phosphatase (PTP) containing domain (Figure 1B). Thus, it is plausible that YipA is specifically processed for intracellular trafficking to the outer membrane, secretion, or formation of the Tc protein complex and proper activity. Additional studies are necessary to determine the significance of YipA processing events.

Our data show a significant upregulation of the Tc genes in the flea (Figure 2); however, a functional role for the Tc proteins has not been established. Since an infectious dose of greater than 1,000 bacteria is required to infect ~50% of fleas [25], fleas are often fed on a heavily infected blood meal (~1.0 x 10^8^ – 1 x 10^9^ CFU/mL) to ensure adequate infection. Although these levels of infection are likely seen by fleas feeding on septicemic animals [26,27], fleas may also feed for a shorter duration or on animals with significantly lower numbers of *Y. pestis* in the blood. Under conditions where fewer *Y. pestis* are initially present within the flea, additional *Y. pestis* factors, such as the Tc proteins, may play a more significant role in facilitating survival within the flea and subsequent preventricular blockage and transmission. Thus, we fed fleas on blood containing a low and mid initial dose (~1 x 10^7^ - 1 x 10^8^) of wild-type KIM6+ or KIM6+Δ*yitA-yipB*. However, even at the lowest initial infectious dose, there were no significant differences between KIM6+ and KIM6+Δ*yitA-yipB* (Table 1), demonstrating that the Tc proteins are not essential for survival within the flea or for normal proventricular blockage. This is consistent with observations made from fleas infected with a blood meal containing ~1.7 x 10^8^ CFU/mL of the KIM6+Δ*yitR* mutant [9]. Thus, the *Y. pestis* Tc proteins are not essential for survival within or to produce a normal transmissible infection in the Oriental rat flea *X. cheopis*. However, it is possible that the Tc proteins are important in survival within or transmission from other flea species.

Although we were unable to detect any phenotype in the flea, we were able to localize YitA and YipA to the outer membrane (Figure 6A) and YitA to the surface (Figure 6B) of *Y. pestis*. Thus, they could play a role in infectivity in the mammalian host after transmission. Although the significance of this is yet to be determined, *Y. pestis* from fleas are resistant to phagocytosis and killing by murine and human neutrophils [5,28], and the Tc proteins were implicated in resistance of *Y. pestis* isolated from fleas to phagocytosis by macrophages [9]. Furthermore, the Tc proteins (protein chimeras and full length YipB) were secreted into culture supernatant, Sf9 cells, RAW macrophages, and HeLa cells in a T3SS-dependent manner [18]. However, *Y. pseudotuberculosis* TcdB protein was detected in both 28 and 37°C culture supernatants [16], indicating a T3SS-independent mechanism of Tc protein secretion. Although we saw minimal production of YitA and YipA after prolonged growth at 37°C, they persisted for several hours after temperature upshift. Therefore, it is plausible that *Y. pestis* Tc proteins produced by *Y. pestis* while in the flea are translocated into mammalian host cells upon transmission, where they act to disrupt the host immune response.

## Conclusion

*Y. pestis* encodes homologues to the *P. luminescens* insecticidal toxins which are highly expressed within the flea vector. However, our data show that *Y. pestis* Tc proteins, unlike *P. luminescens* toxins [2], are not toxic to fleas and are not essential for survival within the flea midgut or in blockage of the proventriculus. Thus, our data indicate that *Y. pestis* Tc proteins have evolved to limit toxicity to their insect vector. Although the *Y. pestis* Tc proteins may play a yet unidentified important role in survival in the environment, the fact that high levels of YitA and YipA protein are produced by *Y. pestis* while in the flea, and that YitA was identified on the bacterial surface, in addition to other evidence to date [2,9,16], suggests that they are more active against mammalian than insect cells. Thus, *Y. pestis* Tc proteins may have evolved to play a role in subversion of the mammalian immune response, plausibly through resistance to phagocytic cells of the innate immune system or in intracellular survival. Furthermore, our data suggest that since the *Y. pestis* Tc proteins are minimally produced after growth in culture compared to growth in the flea, virulence studies to date using *Y. pestis* grown in broth are inadequate to determine the contribution of Tc proteins, and other proteins specifically upregulated during growth in the flea, in transmission and virulence. Thus, experiments using *Y. pestis* over-producing YitR are underway to determine if the Tc proteins play a role in pathogenicity. Additionally, experiments to determine if *Y. pestis* Tc proteins are secreted or translocated into host neutrophils via the T3SS and their effect on neutrophil phagocytosis and killing are being performed.

## Methods

### Bacterial strains, plasmids and culture conditions

Strains and plasmids used are listed in Table 2. All primers used are listed in Table 3. All experiments were performed under Biosafety Level 2 containment using avirulent *Y. pestis* KIM6+ strains which lack the pCD1 (Lcr) virulence plasmid and are excluded from CDC Category A Select Agent rules. All transformants were created with approval from the Rocky Mountain Laboratories Institutional Biosafety Committee using approved antibiotic resistance genes. Where indicated, the low-copy plasmid pWKS130::*yitR*[9] or the high-copy plasmid pCR-XL-TOPO::*yitR*, created by cloning the PCR-amplified YitR open reading frame flanked by ~300 bp of upstream and downstream sequence into pCR-XL-TOPO (Life Technologies, Grand Island, NY), was also added to *Y. pestis* to increase YitA and YipA synthesis under broth culture conditions. KIM6+Δ*yitA-yipB* (Figure 1A) was created using the lambda red recombinase-mediated knockout procedure described previously [29]. To create both YitA- and YipA-β-lactamase translational fusions, intermediate pUC19 plasmids which contained the terminal 500 bp of the *yitA* or *yipA* open reading frame (minus the stop codon) ligated to the 500 bp immediately downstream of the stop codon were created. Primers used to amplify these regions prior to cloning were flanked with XbaI and XhoI or XhoI/SalI and SphI restriction enzyme sites (Table 3). Following digestion with the appropriate enzymes, the pair of PCR products was cloned into XbaI- and SphI-digested pUC19 in a three-way ligation, resulting in recombinant *yitA* or *yipA* sequence in which the stop codon was replaced by a 12-nt sequence containing XhoI and SalI restriction sites. The mature domain of TEM-1 β-lactamase (lacking the N-terminal signal sequence that directs β-lactamase to the periplasm but including the stop codon) was amplified from pBR322 using primers flanked with XhoI and SalI sites. This fragment was then inserted into both recombinant pUC19 plasmids, resulting in plasmids that contained translational fusions of the YitA or YipA termini with β-lactamase, linked by the 6-nt XhoI sequence (introducing the 2 additional amino acids Leu and Glu) and flanked by 500 nt of *yitA* or *yipA* downstream sequence following the β-lactamase stop codon. These constructs were digested from pUC19 using XbaI and SphI, gel purified, and ligated into the suicide vector pDS132 [30]. Recombinant pDS132 plasmids containing *yitA*- or *yipA*-β-lactamase were placed into *Escherichia coli* S17-1 and transferred from *E. coli* S17-1 to *Y. pestis* via conjugation. Transconjugants were selected on *Yersinia* selective agar [31] with chloramphenicol, and verified by PCR. After overnight growth in brain heart infusion (BHI) broth without selection, transconjugants were placed on BHI agar containing 5% sucrose to select for allelic exchange mutants [32], which were further screened for chloramphenicol sensitivity and verified by PCR and Western blot analysis using anti-YitA, anti-YipA, and anti-β-lactamase antibodies (Millipore, Billerica, MA). *Y. pestis* was grown in BHI broth at 22°C overnight from frozen stocks and subcultured into fresh BHI at 22°C twice prior to each assay. Where appropriate, kanamycin (30 μg/mL), carbenicillin (100 μg/mL), or chloramphenicol (10 μg/mL) were added to the broth cultures at the indicated final concentration.

**Table 2:** ***Y. pestis *strains and plasmids used in this study**

**Strain or Plasmid**	**Description**^***a***^	**Source or reference**
Strains		
KIM6+	Tc genes present, pCD1-negative	[33]
KIM6+Δ*yitR*	Deletion of the regulator YitR	[9]
KIM6+Δ*yitA-yipB*	Deletion of the Tc locus from YitA to YipB, YitR still present	This study
KIM6+ (pCR-XL-TOPO::*yitR*)	KIM6+ transformed with a high-copy plasmid producing YitR (Kan, Zeo)	This study
KIM6+Δ*yitA-yipB* (pCR-XL-TOPO::*yitR*)	KIM6+Δ*yitA-yipB* transformed with a high-copy plasmid producing YitR (Kan, Zeo)	This study
KIM6+ (pWKS130::*yitR*)	KIM6+ transformed with a low-copy plasmid producing YitR (Kan)	This study
KIM6+Δ*yitA-yipB* (pWKS130::*yitR*)	KIM6+Δ*yitA-yipB* transformed with a low-copy plasmid producing YitR (Kan)	This study
KIM6+ YitA-β-lactamase	YitA::β-lactamase translational fusion	This study
KIM6+ YipA-β-lactamase	YipA::β-lactamase translational fusion	This study
KIM6+ YitA-β-lactamase (pCR-XL-TOPO::*yitR*)	KIM6+ YitA-β-lactamase transformed with a high-copy plasmid producing YitR (Kan, Zeo)	This study
KIM6+ YipA-β-lactamase (pCR-XL-TOPO::*yitR*)	KIM6+ YipA-β-lactamase transformed with a high-copy plasmid producing YitR (Kan, Zeo)	This study
KIM6+ (pCR-XL-TOPO::*yitR*) (pAcGFP1)	KIM6+ (pCR-XL-TOPO::*yitR*) transformed with a plasmid producing GFP (Kan, Zeo, Amp)	This study
KIM6+Δ*yitA-yipB* (pCR-XL-TOPO::*yitR*) (pAcGFP1)	KIM6+Δ*yitA-yipB* (pCR-XL-TOPO::*yitR*) transformed with a plasmid producing GFP (Kan, Zeo, Amp)	This study
Plasmids		
pCR-XL-TOPO::*yitR*	*yitR* expressed under native promoter from a high-copy plasmid (Kan, Zeo)	This study
pWKS130::*yitR*	*yitR* expressed under native promoter from a low-copy plasmid (Kan)	[9]
pAcGFP1	Constitutively producing GFP (Amp)	Clontech
pUC19	Cloning plasmid for creation of YitA- and YipA-β-lactamase fusion constructs (Amp)	[34]
pDS132::*yitA*-β-lactamase	Suicide plasmid containing YitA-β-lactamase fusion construct (Cam)	This study
pDS132::*yipA*-β-lactamase	Suicide plasmid containing YipA-β-lactamase fusion construct (Cam)	This study
pET300/NT-DEST::*yitA*	Production of YitA with an N-terminal His tag (Amp)	This study
pET300/NT-DEST::*yipA*	Production of YipA with an N-terminal His tag (Amp)	This study

*a* antibiotic resistances where present are noted in parentheses: *Kan* = Kanamycin, *Amp* = Ampicillin, *Cam* = Chloramphenicol, *Zeo* = Zeocin.

**Table 3:** **Primers used for *yitR *amplification, creation of KIM6+Δ*yitA-yipB, *and creation of YitA and YipA-β-lactamase fusion proteins**

**Primer Designation**	**Sequence**^***a***^
YitR Forward Primer	5^′^-AGTTGAGCTCGTCTGCATTGATTATTTGACC-3^′^
YitR Reverse Primer	5^′^-AGTTTCTAGAGATCGTTGCGTAGCTGTGTTGC-3^′^
YitA-YipB KO Primer Forward	5^′^-TGGCATCAATAAACTGGCCTTTTCTGTTGCAC
	CAAAAATATGTGTAGGCTGGAGCTGCT-3^′^
YitA-YipB KO Primer Reverse	5^′^-TTCCCTATTCAAAATAGGGAAGGTGTTTAAAA
	TTAATAAACATATGAATATCCTCCTTA-3^′^
YitA C-terminus Forward	5^′^-ATCTCTAGACCCCAGAACACCCCATGTAT-3^′^
YitA C-terminus Reverse	5^′^-GATCTCGAGAAGATTAACTCTTAGCTTGT-3^′^
YitA Downstream Forward	5^′^-ATCCTCGAGGTCGACAAAAACGTTATTACATCCAA-3^′^
YitA Downstream Reverse	5^′^-GATGCATGCCTGATTTGACTGATTTTTCC-3^′^
YipA C-terminus Forward	5^′^-ATCTCTAGAAGAGACTTAAAGCATATGGT-3^′^
YipA C-terminus Reverse	5^′^-GATCTCGAGTCGATTCTGTTTGCTATATA-3^′^
YipA Downstream Forward	5^′^-ATCCTCGAGGTCGACTTAAGAATATTAAGGAGCCA-3^′^
YipA Downstream Reverse	5^′^-GATGCATGCTGGCCGTTCAGGTTGCAGTT-3^′^
Mature β-lactamase Forward	5^′^-ATCCTCGAGCACCCAGAAACGCTGGTGA-3^′^
Mature β-lactamase Reverse	5^′^-GATGTCGACTTACCAATGCTTAATCAGTGA-3^′^

*a* Underlined sequence denotes restriction enzyme site addition.

### YitA and YipA purification and antibody generation

YitA and YipA were cloned from the *Y. pestis* KIM Gateway Entry Clone Library created by the Pathogen Functional Genomics Resource Center at the Institute for Genomic Research (TIGR, Rockville, MD). Clones encoding YitA (y0183, TIGR sequence id: 37681/clone id: 141008) and YipA (y0190, sequence id: 37674/clone id: 140911) were used. The *yitA* and *yipA* genes were cloned into Champion pET300/NT-DEST vector (Life Technologies) and electroporated into *E. coli* BL21 (Life Technologies). Production of YitA and YipA after IPTG induction and 4 hours of growth at 37°C was verified by SDS-PAGE and by Western blot using anti-6-His antibody (Covance, Princeton, NJ). YitA and YipA proteins were separated by SDS-PAGE and the appropriate-sized bands were excised from the gel, electroeluted and concentrated by centrifugation at 3,200 x g in centrifugal filters (Amicon Ultra Ultracel 3 K, Millipore). Eluted proteins were further purified by affinity chromatography on nickel-nitrilotriacetic acid (Ni-NTA) resin columns (Qiagen Inc., Valencia, CA). Rabbit polyclonal antiserum was generated against purified YitA (anti-YitA) and YipA (anti-YipA) (Lampire Biological Laboratories, Inc., Pipersville, PA). Non-specific antibodies present in the sera were removed by absorption with *Y. pestis* KIM6+Δ*yitA-yipB* cells [35].

### Flea infections and determination of proventricular blockage

All animals were handled in strict accordance with good animal practice as defined by NIH animal care and use policies and the Animal Welfare Act, USPHS; and all animal work was approved by the Rocky Mountain Laboratories (RML) Animal Care and Use Committee. Fresh mouse blood was obtained from adult RML Swis-Webster mice by cardiac puncture. *X. cheopis* fleas were allowed to feed on an infected blood meal containing ~1 x 10^7^ to ~1 x 10^8^ CFU/mL of *Y. pestis* KIM6+Δ*yitA-yipB* or KIM6+ in 5 mL of fresh heparinized mouse blood*.* For each infection, 95 female fleas and 55 male fleas that had taken a blood meal were selected. Samples of 20 female fleas were collected immediately after infection (day 0) and at 7 and 28 days postinfection and stored at −80°C. Throughout the 28 days following infection, fleas were maintained at 22°C and fed twice weekly on normal uninfected mice. Immediately after each feeding, fleas were checked by microscopy for blockage of the proventriculus as previously described [4,36]. Fleas stored at −80°C were later surface sterilized and individually triturated and plated to determine *Y. pestis* infection rate and mean bacterial load per infected flea as previously described [4].

### Western blot analysis of YitA and YipA levels in fleas and liquid media

2 to 4 weeks after an infectious blood meal containing 2 x 10^9^*Y. pestis*/mL, flea midguts were dissected and pooled in lysing matrix H tubes (MP Biomedicals, Solon, OH) with 1 mL Dulbecco’s phosphate-buffered saline (DPBS). Tubes containing infected flea midguts were placed in a FastPrep FP120 (Qbiogene, Inc., Carlsbad, CA) homogenizer for 15 s to triturate midguts and disrupt bacterial aggregates.

Production of YitA and YipA at different temperatures in BHI, heparinized whole sheep blood (Quad Five, Ryegate, MT), or RPMI 1640 (Life Technologies) cell culture media was assessed by subculturing 22°C overnight cultures (1:10 ratio for 10°C culture and 1:20 ratio for all other temperatures) into each medium and incubating overnight at 10°C, 22°C, 28°C or 37°C. All cultures had an OD 600 nm between 1.2 and 2.0 prior to processing.

Persistence of YitA and YipA following transfer of *Y. pestis* grown at 22°C to 37°C was assessed by taking 100 mL overnight BHI cultures of KIM6+ (pCR-XL-TOPO::*yitR*) or KIM6+Δ*yitA-yipB* (pCR-XL-TOPO::*yitR*) grown at 22°C and transferring them to 37°C. A 100 mL culture of KIM6+ (pCR-XL-TOPO::*yitR*) was kept at 22°C as a positive control. Samples were taken from the cultures 1 to 30 h after transfer.

For Western blot analysis, all bacteria were pelleted, washed, resuspended in DPBS and quantified by Petroff-Hausser direct counts. Samples were normalized to equivalent cell numbers and the lysates of approximately 3 ×10^7^ bacteria (grown in broth or isolated from fleas) were separated by SDS-PAGE in lanes of 4-15% precast polyacrylamide gels (Criterion TGX, Bio-rad, Hercules, CA). Samples were then transferred to 0.2 μm nitrocellulose for Western blot analysis. YitA and YipA were detected using anti-YitA or anti-YipA serum. Mouse antiserum against the constitutively expressed *Y. pestis* outer membrane protein Ail [37] was used for a sample loading control. Goat anti-rabbit IgG or goat anti-mouse IgG antibodies conjugated to alkaline phosphatase (Life Technologies) and BCIP/NBT-Blue liquid substrate (Sigma-Aldrich, St. Louis, MO) were used to visualize protein bands.

### Fractionation of *Y. pestis*

*Y. pestis* was grown overnight in BHI at 22°C and subcultured into 500 mL of fresh BHI at a 1:100 ratio. Cultures were grown overnight with aeration at 22°C. Bacteria were pelleted, washed, and the cytoplasmic, periplasmic, cytosolic membrane, and outer membrane fractions were collected using a previously described protocol [38]. The total protein concentration of the fractions was determined (Qubit Fluorometer Protein Assay Kit, Life Technologies) and normalized to 1.0 mg/mL of total protein. For Western blot analysis, 30 μg of each fraction was loaded per well.

### Immunofluorescence microscopy

*Y. pestis* KIM6+ (pCR-XL-TOPO::*yitR*) (pAcGFP1), or KIM6+Δ*yitA-yipB* (pCR-XL-TOPO::*yitR*), (pAcGFP1) as a negative control, were grown overnight in BHI at 22°C. Bacteria were pelleted and washed two times and resuspended in PBS. Bacteria were added to glass coverslips in 24-well microtiter plates and centrifuged at 3,000 x g for 10 min. Bacteria were fixed in 4% paraformaldehyde for 15 min at 37°C and washed. Bacteria were incubated with anti-YitA or anti-YipA rabbit serum for 30 min at 37°C, washed, stained with Alexa Fluor 568 goat anti-rabbit IgG (Life Technologies), and imaged by fluorescence microscopy. Pictures were taken using a Photometrics CoolSnap HQ black and white camera and images were artificially colored and combined using MetaMorph software version 7.5.6.0 (Molecular Devices, Sunnyvale, CA).

## Competing interests

The author(s) declare that they have no competing interests.

## Authors’ contributions

JLS and BJH wrote the manuscript. JLS, COJ, DLL, CMC and BJH conceived of and participated in the design of the study. JLS, COJ, and BJH performed the experiments. COJ created *Y. pestis* KIM6+Δ*yitR*. DLL created *Y. pestis* KIM6+Δ*yitA-yipB*. SIM, CMC, and BJH provided materials and reagents. All authors read and approved the final manuscript.
